# Approximate Measurement Invariance of Willingness to Sacrifice for the Environment Across 30 Countries: The Importance of Prior Distributions and Their Visualization

**DOI:** 10.3389/fpsyg.2021.624032

**Published:** 2021-07-22

**Authors:** Ingrid Arts, Qixiang Fang, Rens van de Schoot, Katharina Meitinger

**Affiliations:** Department of Methodology and Statistics, Faculty of Social Sciences, Utrecht University, Utrecht, Netherlands

**Keywords:** measurement invariance, visualization, Bayes, group ranking, MGCFA, prior sensitivity, Bayesian approximate measurement invariance (BAMI)

## Abstract

Nationwide opinions and international attitudes toward climate and environmental change are receiving increasing attention in both scientific and political communities. An often used way to measure these attitudes is by large-scale social surveys. However, the assumption for a valid country comparison, measurement invariance, is often not met, especially when a large number of countries are being compared. This makes a ranking of countries by the mean of a latent variable potentially unstable, and may lead to untrustworthy conclusions. Recently, more liberal approaches to assessing measurement invariance have been proposed, such as the alignment method in combination with Bayesian approximate measurement invariance. However, the effect of prior variances on the assessment procedure and substantive conclusions is often not well understood. In this article, we tested for measurement invariance of the latent variable “willingness to sacrifice for the environment” using Maximum Likelihood Multigroup Confirmatory Factor Analysis and Bayesian approximate measurement invariance, both with and without alignment optimization. For the Bayesian models, we used multiple priors to assess the impact on the rank order stability of countries. The results are visualized in such a way that the effect of different prior variances and models on group means and rankings becomes clear. We show that even when models appear to be a good fit to the data, there might still be an unwanted impact on the rank ordering of countries. From the results, we can conclude that people in Switzerland and South Korea are most motivated to sacrifice for the environment, while people in Latvia are less motivated to sacrifice for the environment.

## Introduction

One of the main issues the world population faces today is climate and environmental change. Some of the challenges that have to be faced include floods, droughts, food insecurity, and biodiversity loss. These challenges may give rise to socioeconomic problems such as refugee crises, relocating populations and cities, and famines (Zhang et al., 2020). As the challenges will differ across regions, but are not limited by national borders, international cooperation is required. At the same time, a “one size fits all” solution is unlikely to solve these issues (Andonova and Coetzee, 2020). Several studies have been conducted on how the inhabitants of different countries perceive the subject of climate and environmental change, and the different aspects of social behavior regarding this subject: e.g., knowledge of climate change, risk perception, and the willingness to act (van Valkengoed and Steg, 2019). Hadler and Kraemer (2016) showed that the inhabitants of different countries do not assess all these threats in the same way: in some countries air pollution is seen as a major threat, while in others water shortages are considered a hazard.

The term “environmental concern” has been used widely to explain environmental behavior (e.g., Dunlap and Jones, 2002; Bamberg, 2003; Schultz et al., 2005; Franzen and Meyer, 2010; Marquart-Pyatt, 2012a; Fairbrother, 2013; Pampel, 2014; Mayerl, 2016; Pisano and Lubell, 2017; Shao et al., 2018). However, a clear definition of this concept is lacking (e.g., Dunlap and Jones, 2002; Schultz et al., 2005). Bamberg (2003, p. 21) described environmental concern as “the whole range of environmentally related perceptions, emotions, knowledge, attitudes, values, and behaviors,” while Dunlap and Jones (2002, p. 485) described environmental concern as “the degree to which people are aware of problems regarding the environment and support efforts to solve them and/or indicate the willingness to contribute personally to their solution.” Following the latter definition, environmental concern consists of at least two parts: on the one hand, perceptions of environmental problems (e.g., risks and beliefs), and, on the other hand, the willingness to contribute to the solution (e.g., to pay more taxes or higher prices, or to fly less). This translates into two latent variables that operationalize environmental concern: “environmental attitude” (EA) and “willingness to sacrifice (or pay) for the environment” (WTS). These two latent variables have been used both individually and in combination to operationalize environmental concern (Mayerl and Best, 2019). The latent variable WTS is frequently used to measure the extent to which people are willing to sacrifice something in their daily life (money, goods, time, comfort) to save the environment, and has been examined by several authors (e.g., Ivanova and Tranter, 2008; Fairbrother, 2013; Franzen and Vogl, 2013; Pampel, 2014; Sara and Nurit, 2014; Shao et al., 2018). The relation with cultural, sociological, economic, or political factors has been studied quite extensively (e.g., Marquart-Pyatt, 2012b; Franzen and Vogl, 2013; Pampel, 2014; Bozonnet, 2016; McCright et al., 2016; Shao et al., 2018).

Large-scale surveys are often used for exploring knowledge, attitudes, and (intentional) behavior regarding climate and environmental change (e.g., Bamberg, 2003; Franzen and Meyer, 2010; Marquart-Pyatt, 2012a; Hadler and Kraemer, 2016; Knight, 2016; Pisano and Lubell, 2017; Libarkin et al., 2018). One precondition for the valid comparison of attitudes toward climate and environmental change across many countries is that measurement properties are equivalent across countries (Jöreskog, 1971; Vandenberg and Lance, 2000). This means that all participants in all countries should interpret both the survey questions and the underlying latent variables in the same way. This equivalence of measurement properties is also called Measurement Invariance (MI). Establishing whether MI holds is usually done by conducting a maximum-likelihood (ML) Multi-Group Confirmatory Factor Analysis (MGCFA). There are at least four types of MI: configural (also referred to as “weak”), metric, scalar (“strong”), and residual (“strict”) invariance. Configural invariance allows for the comparison of latent variables among groups, metric invariance allows for a comparison of the items (questions) that make up the latent variable(s) among groups, and scalar invariance allows for the comparison of latent means across groups. Scalar invariance, however, is rarely established, especially when many groups are compared (e.g., Muthen and Asparouhov, 2013; Lommen et al., 2014; Kim et al., 2017; Marsh et al., 2018)^1^.

Measurement invariance of the latent variable WTS has been investigated by Mayerl and Best (2019), and they established both configural and metric invariance, but not scalar invariance. Using ML MGCFA, Marquart-Pyatt (2012b) also found configural and metric invariance, but not scalar invariance. To our knowledge, scalar invariance for the latent variable WTS has not been found by other authors, rendering the substantive interpretation of results from country rankings potentially untrustworthy (Byrne and van de Vijver, 2017; Marsh et al., 2018).

Alternative approaches have been proposed, such as alignment optimization, which allows for few but larger parameters differences between some groups (Asparouhov and Muthén, 2014), Bayesian Approximate MI (Muthén and Asparouhov, 2012; van de Schoot et al., 2013), hereinafter referred to as BAMI^2^, which allows multiple but small differences between all groups, or a combination of both, BAMI alignment (Asparouhov and Muthén, 2014). When BAMI alignment is used, small variances are allowed for each group, while a few groups are allowed to have large variances. This leads to fewer noninvariant parameters than when the ML alignment method is applied, facilitating the interpretation of the model (Asparouhov and Muthén, 2014). Although this might be a highly interesting approach when a comparison of many groups is desired, it seems that, at least up until now, this approach has not been applied often: we only found two studies in which BAMI and alignment are combined: De Bondt and Van Petegem (2015) and van de Vijver et al. (2019), and certainly not in the field of environmental change.

The key to using Bayesian methods is the use of priors: some “wiggle room” is defined between which the variances of different groups are allowed to vary. However, the selection of these priors (from simulation studies, literature, or experience) is not an easy task. It seems that researchers applying Bayesian methods are not always fully aware of the potential impact of specifying priors (e.g., Spiegelhalter et al., 2000; Rupp et al., 2004; Ashby, 2006; Kruschke et al., 2012; Rietbergen et al., 2017; van de Schoot et al., 2017; König and van de Schoot, 2018; Smid et al., 2020). Nonetheless, for the verification and reproducibility of research (Munafò et al., 2017; van de Schoot et al., 2021), it is crucial to evaluate the influence of varying priors on the impact of substantive conclusions, which is referred to as sensitivity analysis. Some general guidelines regarding prior sensitivity can be found in the literature (e.g., Depaoli and van de Schoot, 2017; van Erp et al., 2018; van de Schoot et al., 2019; Pokropek et al., 2020). Although a sensitivity analysis of different prior settings helps to determine the impact of prior variances on substantive conclusions, it has, to our knowledge, never been applied for BAMI with empirical data.

The goals of our article are to apply the method of BAMI to the concept of “willingness to sacrifice (or pay) for the environment,” compare the results of different prior settings to each other and to other methods of dealing with measurement invariance (i.e., ML MGCFA and the ML alignment method) through visualization, and to provide an example for a transparent workflow.

In what follows, we first provide a technical introduction to the four methods we used to assess MI. As it can be difficult to interpret multiple models and methods, and because we want to be as transparent as possible in our decision-making process, we summarize our design choices and possible alternatives in a decision tree. We test the models to evaluate whether and how different prior variances influence the ranking of the countries on the latent variable WTS. We visualize the results to facilitate a comparison of the latent means of different models and methods without the use of complex and elaborate tables. All appendices, the scripts to reproduce our results, the final output files and additional material can be found on website of the Open Science Framework (OSF) (Arts et al., 2021).

## Technical Background

In this section, we introduce the four methods we used to evaluate measurement invariance: (1) ML MGCFA, (2) ML MGCFA using the alignment optimization, (3) BAMI, and (4) BAMI in combination with the alignment method.

###  MGCFA

The MGCFA model is defined as:

(1) $$\begin{array}{r} y_{ipg}=\nu_{pg}+{\text{λ}}_{pg}\eta_{ig}+\epsilon_{ipg} \end{array}$$

where *p* = 1, ...*P* is the number of observed indicator variables, *g* = 1, ...*G* is the number of groups, *i* = 1, ...*N* is the number of individual observations, λ_*pg*_ is a vector of factor loadings, *ν*_*pg*_ is a vector of intercepts and *η*_*ig*_ is a vector of latent variables. Furthermore, *ϵ*_*ipg*_ is a vector of error terms that is assumed to be normally distributed with *N*(0, *θ*_*pg*_), and *η*_*ig*_ is assumed to have a distribution of *N*(*α*_*g*_, *φ*_*g*_). *θ*_*pg*_ is the variance of *ϵ*_*ipg*_, *α*_*g*_ is the mean of normally distributed latent variable *η*_*ig*_, and *φ*_*g*_ is the variance of *η*_*ig*_. For WTS let P = 3 (3 items) and G = 30 (30 countries), which means that λ_*pg*_ is a 3 × 30 matrix. The same is true for *ν*_*pg*_.

In the configural model, both λ and *ν* are allowed to vary across groups^3^, but the factor structure is equal for all groups, that is, in all 30 countries the latent variable WTS is covered by the same three items.

When both the number of latent variables and the factor loading λ are held equal across groups but the intercept *ν* is allowed to vary, one is testing for metric invariance: λ_11_ = λ_12_ = λ_13_, etc. This means that for every group, the latent variable *η*_*g*_ contributes equally to item *y*_*pg*_.

If metric invariance holds, it is possible to test for scalar invariance. In this case both loadings λ and intercepts *ν* are held equal across groups: λ_11_ = λ_12_ = λ_13_ etc. and *ν*_11_ = *ν*_12_ = *ν*_13_ etc., so that Equation (1) becomes:

(2) $$\begin{array}{r} y_{p}=\nu_{p}+{\text{λ}}_{p}\eta +\epsilon_{ipg} \end{array}$$

When scalar invariance holds, the latent means of WTS can be compared between groups, and a ranking of the latent means can be made. However, scalar, or strong, invariance is very rare, especially when comparing many groups (Asparouhov and Muthén, 2014; Byrne and van de Vijver, 2017; Kim et al., 2017; Marsh et al., 2018). This is due to the fact that with increasing number of countries, the probability increases that countries substantially deviate in answering behavior. When many groups with small deviations are being compared, these small deviations add up to the non-invariance of the scale assessing WTS.

###  Alignment Optimization

To reduce the impact of a lack of measurement invariance for many groups, the alignment optimization method has been introduced (Muthen and Asparouhov, 2013; Asparouhov and Muthén, 2014). Alignment optimization consists of two steps (Asparouhov and Muthén, 2014). First, a null model M_0_ is estimated with loadings and intercepts allowed to vary across groups. As loadings and intercepts are freed across groups, factor means and factor variances are set to 0 and 1 for every group: *α*_*g*_ = 0 and *φ*_*g*_ = 1. Now, the latent variable for the null model *η*_*g*0_ can be calculated.

Second, the method divides groups *G* into pairs *Q* and tries to find, for every *Q*, the intercepts and loadings that yield the same likelihood as the M_0_ model (Asparouhov and Muthén, 2014; Flake and McCoach, 2018). Now, λ_*pg*_ and *ν*_*pg*_ can be calculated, where *α*_*g*_ and *φ*_*g*_ have to be chosen in such a way that they minimize the amount of measurement non-invariance and *q*1, *q*2, etc. are the different pairs of groups in the data. For the full set of equations, see Asparouhov and Muthén (2014); Flake and McCoach (2018). This means that, for the latent variable WTS, *q* = 1...435 for every item (for every item there are 435 possible pairs).

The total amount of measurement non-invariance is shown by the total loss/simplicity function *F*:

(3) $$\begin{array}{l} F=\sum_{p}\sum_{g_{1}<g_{2}}w_{g_{1},g_{2}}f({\text{λ}}_{pg_{1},q_{1}}-{\text{λ}}_{pg_{2},q_{1}})\\ \;+\sum_{p}\sum_{g_{1}<g_{2}}w_{g_{1},g_{2}}f(\nu_{pg_{1},q_{1}}-\nu_{pg_{2},q_{1}}) \end{array}$$

In Equation (3), for the intercepts and loadings of every *Q*, the differences between the parameters are summed and then scaled by the Component Loss Function (CLF) *f*. Group sizes are appointed by weight factors *w*_*g*_1__ and *w*_*g*_2__, where *w*_*g*_1__ is the weight factor of group 1 and *w*_*g*_2__ is the weight factor of the, differently sized group 2. In this way, bigger pairs of groups contribute more to the total loss function than smaller pairs. The weight factor can be calculated as follows:

(4) $$\begin{array}{r} w_{q}=w_{g_{1},g_{2}}=\sqrt{N_{g_{1}}N_{g_{2}}} \end{array}$$

The CLF has been used in exploratory factor analysis (EFA) to estimate factor loadings with the simplest possible structure (Jennrich, 2006). For the alignment the CLF is:

(5) $$\begin{array}{r} f\left(x\right)=\sqrt{\;}\sqrt{x^{2}+\epsilon } \end{array}$$

with *ϵ* being a small number, for example, 0.01 (Asparouhov and Muthén, 2014). This positive number ensures that *f*(*x*) has a continuous first derivative, making the optimization of the total loss function *F* easier. As *ϵ* is so small, $f\left(x\right)\approx \sqrt{\left|x\right|}$, which leads to no loss if *x* = 0, amplified loss if *x* < 1, and attenuated loss if *x* > 1 (Asparouhov and Muthén, 2014). Due to this CLF, *F* will be minimized when there are a few large non-invariant loadings and intercepts and a majority of approximately non-invariant loadings and intercepts (Kim et al., 2017). When there are many medium-sized non-invariant parameters, the total loss function does not optimize (Flake and McCoach, 2018). If *F* does optimize, the parameters *α*_*g*_ and *φ*_*g*_ will be identified for all groups except the first one. For the first group, the variance can be calculated using the following parameter constraints, making the number of estimated parameters (2*G* − 1):

(6) $$\begin{array}{r} \varphi_{1}\times \ldots \times \varphi_{g}=1 \end{array}$$

*α*_1_ can be set to 0, although this is not always needed and might lead to untrustworthy estimates (Asparouhov and Muthén, 2014). When *α*_1_ and *φ*_1_ are both constrained, the alignment is called FIXED in Mplus, and when only *φ*_1_ is constrained, that alignment is said to be FREE.

Although the alignment optimization allows for some large invariances between groups, all other groups are assumed to have the same loadings and intercepts. In other words, small variances between groups cannot be taken into account. To ensure that mean and variance can be fixed for one country (as it is in the MGCFA and BAMI), we have to opt for the FIXED alignment and specify one country to be fixed.

###  BAMI

A synopsis of Bayesian statistics, including the most important aspects of determining prior distributions, likelihood functions and posterior distributions, in addition to discussing different applications of the method across disciplines can be found in van de Schoot et al. (2021).

With BAMI, priors with a mean of zero and some small variance are put on the differences between factor loadings and the differences between intercepts across groups: the terms λ_*pg*_ and *ν*_*pg*_ from Equation (1) are now estimated being approximately equal across groups instead of exactly equal: λ_11_ ≈ λ_12_ ≈ λ_13_ etc. instead of λ_11_ = λ_12_ = λ_13_, etc. and *ν*_11_ ≈ *ν*_12_ ≈ *ν*_13_ etc. instead of *ν*_11_ = *ν*_12_ = *ν*_13_, etc.

The prior is not put directly on the differences between parameters, but on the covariances between parameters. This means that, for instance

(7) $$\begin{array}{r} V\left({\text{λ}}_{11}-{\text{λ}}_{12}\right)=V\left({\text{λ}}_{11}\right)+V\left({\text{λ}}_{12}\right)-2Cov\left({\text{λ}}_{11},{\text{λ}}_{12}\right) \end{array}$$

where *V*(λ_11_ − λ_12_) is the difference between the variances of the first loading of the first group and the first loading of the second group. If we assume that these prior variances are small, for instance 0.5, and the covariance is 0.495, that would lead to a value of 0.01 for *V*(λ_11_ − λ_12_), or *V*^*d*^.

BAMI uses strong informative priors on cross-group variances of loadings λ and intercepts *ν*. It is important to carefully select these priors since they have a strong impact on the posterior results. Large values of *V*^*d*^ will result in decreasing the chance of model convergence, as they do not impose enough information on the model (Muthén and Asparouhov, 2012). Smaller values of *V*^*d*^, on the other hand, might bring the model too close to a scalar model, reducing flexibility of the model to deal with the existing non-invariance.

###  BAMI With Alignment

The alignment method and BAMI can be combined. In that case, small variances are allowed for each group, while a few groups are allowed to have large variances. The alignment method for BAMI is similar to that for the exact method:

In the first step, an M_0_ model is estimated, from which the optimal set of measurement parameters from the configural model is calculated. Now the M_0_ model is a model where the intercepts and loadings are approximately equal across groups and the factor means and variances are estimated as free parameters in all groups but the first one.

In the second step, this M_B0_ model, the posterior of the configural factor loadings and intercepts are computed using the following equations:

(8) $$\begin{array}{r} {\text{λ}}_{pg,0}={\text{λ}}_{pg,B}{\sqrt{\varphi }}_{Bg} \end{array}$$

(9) $$\begin{array}{r} \nu_{pg,0}=\nu_{pg,B}+\alpha_{Bg}{\text{λ}}_{pg,B} \end{array}$$

where λ_*pg*, 0_ and *ν*_*pg*, 0_ are the configural loadings and intercepts and *α*_*Bg*_, *φ*_*Bg*_, λ_*pg, B*_, and *ν*_*pg, B*_ are the BAMI parameters. Using the BAMI parameters and Equations (8) and (9), the configural loadings and intercepts are computed for every iteration. These are then used to form the posterior distribution for λ_*pg*, 0_ and *ν*_*pg*, 0_.

In the third and final step, the aligned estimates are obtained for every iteration using the configural factor loading and intercept values to minimize the simplicity function of Equation (3). The aligned parameter values obtained from one iteration are used as starting values in the next iteration. Finally, the aligned parameter values from all iterations are then used to estimate the aligned posterior distribution as well as the point estimates and the standard errors for the aligned parameters (Asparouhov and Muthén, 2014). This leads to fewer non-invariant parameters than when the ML alignment method is applied, facilitating the interpretation of the model (Asparouhov and Muthén, 2014).

## Methods and Data

###  Data

We used the data from the 2010 Module on Environment of the ISSP (ISSP Research Group, 2019). For the full report on this module, see GESIS (2019). The latent variable WTS consists of three questions, see Table 1 for the exact wording, with answers on a five-point response scale (1 being *very unwilling* and 5 being *very willing*) and a *cannot choose* option for participants who could not or would not answer the question. WTS has, in combination with EA, been tested for MI by Mayerl and Best (2019) to explain the concept “environmental concern” when applied to 30 countries: Austria, Belgium, Bulgaria, Canada, Chile, Croatia, Czech Republic, Denmark, Finland, France, Germany, Great Britain, Israel, Japan, Latvia, Lithuania, Mexico, New Zealand, Norway, Philippines, Russia, Slovakia, Slovenia, South Africa, South Korea, Spain, Sweden, Switzerland, Turkey, and the United States. They found that, although metric invariance was achieved, scalar invariance was not. When we repeated this analysis we came to the same conclusion, for the results of this analysis, see Appendix A in Arts et al. (2021). For simplicity reasons, we only focus on the latent variable WTS, just like Ivanova and Tranter (2008); Fairbrother (2013); Franzen and Vogl (2013); Pampel (2014); Sara and Nurit (2014), and Shao et al. (2018). To further analyze this scale, we first ensured that we used the exact same data from the ISSP 2010 environment module and we followed the identical procedure as in the original study to handle missingness (i.e., listwise deletion—correspondence with author, November 26 2019), resulting in the same sample (*n* = 24,583). For the exact procedure and all code, see Appendix A in Arts et al. (2021). The sample sizes per country ranges from 798 (Iceland) to 3,112 (South Africa) with an average group size of 1,401, see for more details Table 2.

**Table 1:** Exact wording of the questions in WTS.

**Number**	**Question**
Q12a	How willing would you be to pay much higher prices in order to protect the environment?
Q12b	How willing would you be to pay much higher taxes in order to protect the environment?
Q12c	How willing would you be to accept cuts in your standard of living in order to protect the environment?

**Table 2:** Participating countries in the ISSP environmental module.

**Country**	**Sample size**	**Country**	**Sample size**
Argentina	1,130	Lithuania	1,023
Australia	1,946	Mexico	1,637
Austria	1,019	Netherlands	1,472
Belgium (Flanders)	1,142	New Zealand	1,172
Bulgaria	1,003	Norway	1,382
Canada	985	Philippines	1,200
Chile	1,436	Portugal	1,022
Croatia	1,210	Russia	1,619
Czech Republic	1,428	Slovakia	1,159
Denmark	1,305	Slovenia	1,082
Finland	1,211	South Africa	3,112
France	2,253	South Korea	1,576
Germany	1,407	Spain	2,560
Great Britain	928	Sweden	1,181
Iceland	798	Switzerland	1,212
Israel	1,216	Taiwan	2,209
Japan	1,307	Turkey	1,665
Latvia	1,000	United States	1,430
Total			50,437

###  Analytical Strategy

We assessed the measurement invariance of the latent variable WTS by applying four methods for detecting MI: ML MGCFA, the ML alignment optimization, BAMI, and BAMI with alignment optimization. For all analyses, one reference country was selected for which the factor mean and factor variance are held to 0 and 1, respectively (Spain). By fixing the mean and variance for a specific reference country for every model, it is ensured that any differences in outcomes are due to a method and not due to a difference in default settings of the model (for some models by default the parameters are fixed for the first group, while for other models it is the last group). We selected Spain as the reference country since the results presented by Mayerl and Best (2019) indicate that the results for WTS from this country can be seen as “average” within the group of thirty countries.

For the BAMI method, both with and without alignment, we tested the effect of different priors on the models. One way of selecting priors for new data is by using the results of simulation studies. Table 3 shows an overview of simulation studies that have investigated BAMI and the priors that were used. As can be seen from this table, the simulation results are not entirely conclusive: The authors of these articles report that they achieve the best results when using priors with a variance of 0.001, 0.005, 0.01, or 0.05. However, the number of groups, group sizes and invariance criteria in these studies vary, complicating a comparison of the best performing prior variance(s).

**Table 3:** Simulation studies using Bayesian approximate measurement invariance.

**Article**	**Number of groups**	**Group size**	**Prior variance**	**Invariance criteria**
Muthén and Asparouhov, 2012	40	500	0.10, 0.05,	PPP
			0.01	
van de Schoot et al., 2013	2	1,000	0.50, 0.05,	PPP, 95% CI
			0.01, 0.005, 0.0005	
Kim et al., 2017	25, 50	50, 100, 1,000	0.05, 0.001	DIC, PPP, 95% CI, BIC
Lek et al., 2018	2	50, 100, 200, 1000	0.10, 0.05, 0.01, 0.001	95% CI
Shi et al., 2017	2	500	0.10, 0.05, 0.01	PPP, 95%CI
Pokropek et al., 2019	24	1500	0.10, 0.05, 0.01, 0.005	cor, RMSEA, 95%CI
Pokropek et al. (2020)	4, 24, 50	400, 1500, 3,000	0.05, 0.025, 0.01, 0.005, 0.001, 0.000^*^	BIC, DIC, PPP

* *A Bayesian model with a prior variance of 0 is the scalar model*. *PPP, posterior predictive p-value; DIC, deviance information criterion; 95% CI, 95% credibility interval; cor, correlation; RMSEA, root mean square error of approximation; BIC, Bayesian information criterion*.

We also searched for empirical studies in which BAMI was applied to empirical data. In a total of 30 empirical studies, there were 13 in which only one prior was used, and in eight of these 13 studies, no specification was given as to why that specific prior was used. In the 17 studies where multiple priors were tested, three did not provide any information on why these priors were selected. The 14 other studies based the priors used on Muthén and Asparouhov (2012), van de Schoot et al. (2013), Asparouhov et al. (2015), or Seddig and Leitgöb (2018). For more information about these empirical studies and their variances see the additional material (Arts et al., 2021). The most frequently used prior variance in these studies is 0.01, followed by 0.05 as recommended by Muthén and Asparouhov (2012) and van de Schoot et al. (2013), respectively. However, other priors were also included in the different sensitivity analyses, ranging from 0.000000001 to 0.5.

We decided to estimate five different models, with priors with a variance of 0.05–0.01 (decreasing at 0.01 per prior) and three models with priors with variances of 0.001, 0.0005, and 0.0001. This includes the prior variances that are used most often in both simulation and empirical studies. Using such a large number of priors should create a clear overview of the influence of different prior variances on the rank order stability of the countries when ranked on their latent factor means. In addition to priors on the differences between loadings and intercepts, there are also priors on other parameters, such as the residuals. However, we will not discuss these priors in this article and we relied on the Mplus default values which can be found in Muthén and Muthén (2019). To ensure that the chains reached their target distributions, we checked whether all iterations after burn-in met the Gelman-Rubin criterion. Therefore, we set the convergence criterion to a rather strict 0.01 instead of the default 0.05 (Muthén and Muthén, 2019) and the maximum and minimum number of iterations to 100,000 and 40,000, respectively.

For the analysis in this article, we used the software Mplus version 8.4 (Muthén and Muthén, 2019). The results were analyzed using R version 6.3.2 (R Development Core Team, 2017) and MplusAutomation version 0-7.3 (Hallquist and Wiley, 2018) was used for the exchange between the two programs. More information about the analysis and the exact Mplus and R code can be found in Appendix B on Arts et al. (2021).

#### Model Fit

To assess model fit for ML MGCFA, the indices that are most widely used are the χ^2^-value, root mean square error of approximation (RMSEA), comparative fit index (CFI), Tucker-Lewis index (TLI), and the standardized root mean square residual (SRMR) (Gallagher and Brown, 2013, p. 298). When testing for configural invariance cutoff values of CFI ≥ 0.95, TLI ≥ 0.95, RMSEA ≤ 0.06, and SRMR ≤ 0.08 have been proposed by Hu and Bentler (1999). When checking for metric and scalar invariance, relative fit indices are more useful than absolute fit indices (Chen, 2007). These relative fit indices are a comparison of configural with metric and metric with scalar fit indices. Depending on these fit indices, the model for metric invariance can be assumed to perform better or worse than the model for configural invariance (and the same is true for metric and scalar invariance). For sample sizes above 300, ΔRMSEA ≤0.015 and ΔCFI ≤0.01 or ΔSRMR ≤0.03 indicate invariance when moving from the configural to the metric model, and ΔRMSEA ≤0.015 and ΔCFI ≤0.01 or ΔSRMR ≤0.01 indicate noninvariance when moving from the metric to the scalar model (Chen, 2007).

For the alignment method, fit indices have not been specified. Muthen and Asparouhov (2014) propose that the results can be considered trustworthy when no more than 25% of the parameters are non-invariant. However, Kim et al. (2017) have argued that this way the degree and location of non-invariance cannot be taken into account.

When BAMI is used, the model fit may be indicated by the posterior predictive p-value (PPP-value). This value indicates the ratio between the iterations for which the replicated χ^2^ value exceeds the observed χ^2^ value (Pokropek et al., 2020). A PPP-value of 0.50 indicates perfect model fit; a value below 0.50 indicates an underfit of the model, and a value above 0.50 indicates an overfit. Furthermore, the 95% credibility interval (CI) should include 0, preferably with 0 in the middle of the interval (Muthén and Asparouhov, 2012; van de Schoot et al., 2013). As PPP-values decline, the model fits the data less well. However, a specific cutoff value at which the model no longer fits the data is hard to determine. Muthén and Asparouhov (2012) suggest that models with PPP-values lower than 0.10, 0.05, or 0.01. do not fit the data anymore. In the literature, PPP-values above 0.05 are often seen as an indication for good model fit. A drawback of the PPP-value is that it might not identify a model with good fit correctly when using different priors with large sample sizes (Asparouhov and Muthén, 2010, 2019; Hoijtink and van de Schoot, 2018; Hoofs et al., 2018)^4^.

Recently, Bayesian versions of fit indices have been proposed: Bayesian RMSEA (BRMSEA), Bayesian CFI (BCFI), and Bayesian TLI (BTLI) can be computed based on differences between the observed and replicated discrepancy functions (Liang, 2020). These Bayesian fit statistics have been implemented in Mplus version 8.4, making it more convenient to identify good model fit (Asparouhov and Muthén, 2019). The calculation of these fit indices is very similar to that of the fit indices of an exact model, and therefore, the same cutoff values can be used (Asparouhov and Muthén, 2019; Garnier-Villarreal and Jorgensen, 2020). This means that BCFI ≥ 0.95, BTLI ≥ 0.95, and BRMSEA ≤0.06 indicate good model fit. However, just as with the ML models, a combination of cutoff values must be used to indicate good or bad model fit. Other criteria that are being used to determine model fit are the BIC (Schwarz, 1978) and the DIC (Spiegelhalter et al., 2002). These information theoretic indices are less self-explanatory than the other fit indices: when selecting the best performing model from a series of models (the model that fits the data best and is the least complex), the model with the lowest BIC or DIC is preferred. This does not mean that the model with the lowest BIC or DIC is a good fit to the data: it is simply preferable to models with a higher BIC or DIC. Asparouhov et al. (2015) stated that, when sample sizes are large, coupled with a large number of observed indicators, DIC is preferable to BIC and Pokropek et al. (2020) concluded that DIC is a good indicator to identify the preferred prior mean and variance. On the other hand, Hoijtink and van de Schoot (2018) stated that the DIC is not suitable for evaluating models with small priors. This makes the use of the DIC as fit index promising, but also shows that its value should be treated with care. At a minimum, DIC should always be combined with other fit indices.

BAMI with alignment has, similar to the ML alignment method, no guidelines to determine model fit. Both De Bondt and Van Petegem (2015) and van de Vijver et al. (2019) tested a model with multiple small prior variances. De Bondt and Van Petegem (2015) used a prior variance of 0.01 and conducted a sensitivity analyses with prior variances decreasing with a factor 10, and van de Vijver et al. (2019) used a prior variance of 0.05 and conducted a sensitivity analyses with prior variances of 0.001, 0.005, 0.01, 0.05, and 0.1. Both De Bondt and Van Petegem (2015) and van de Vijver et al. (2019) analyzed the alignment part of the model by comparing, for each item, the intercepts and loadings across paired groups. This can be a very laborious process when multiple items and multiple groups are concerned. One could also use the rule of thumb that, to obtain trustworthy results, no more than 25% of the parameters can be invariant, as proposed by Muthen and Asparouhov (2014).

As shown above, there are many different criteria and cut-off values that provide insight into whether a model fits the data. Since there are so many different indicators these cutoff values should be treated with care: fit statistics can be influenced by, e.g., sample size or model complexity (Chen, 2007). Additionally, having one indication of good model fit is not enough to conclude that the model is a good fit to the data, and multiple fit statistics may even contradict each other. This exact point was addressed by Lai and Green (2016), who showed that RMSEA and CFI can contradict each other. Even when there is sufficient evidence that a model is a good fit to the data, this does not necessarily mean that it is the best model.

## Results

###  MGCFA

The fit indices for the metric and scalar MGCFA are shown in Table 4. Since the configural model was saturated, the results are not shown here. Therefore, for the metric model we asses the absolute fit indices instead of the relative fit indices. The metric model shows good fit, with a CFI and TLI of 0.993 and 0.989, respectively. With 0.069 the RMSEA value is above 0.06 but still below 0.08, indicating at least a reasonable fit. The fit indices for the scalar model all point to rejection of the scalar model: ΔRMSEA, ΔSRMR and ΔCFI are well above the cutoff values of 0.015, 0.01, and 0.01 (0.085, 0.057, and 0.065, respectively). Based on these results we conclude that scalar invariance is absent, and that a comparison of the latent variable WTS across countries may not be trustworthy. However, this exact approach could be too strict in its assessment.

**Table 4:** Fit statistics of the MGCFA model.

	**χ^2^ (df)**	**Δ*χ*^2^(Δ df)**	***p*-value**	**RMSEA**	**Δ RMSEA**	**SRMR**	**Δ SRMR**	**CFI**	**Δ CFI**	**TLI**	**Δ TLI**
Configural^*^											
Metric	287.324 (58)		0.00	0.069		0.051		0.993		0.989	
Scalar	2382.434 (116)	2095.110 (58)	0.00	0.154	0.085	0.108	0.057	0.928	0.065	0.944	0.045

* *This model was saturated. df, degrees of freedom; RMSEA, root mean square error of approximation; SRMR, standardized root mean square residual; CFI, comparative fit index; TLI, Tucker-Lewis index. Numbers are absolute*.

### Alignment Optimization

Regarding the alignment optimization, the invariant, and non-invariant parameters are shown in Table 5 with non-invariant parameters bolded and in brackets. Most non-invariant parameters can be found in the intercepts, with 48 non-invariant parameters, while for the loadings only seven parameters are non-invariant. However, a total of 55 parameters are non-invariant, which is 30.55% of all parameters. This is well above 25%, a rough cut-off value proposed by Muthen and Asparouhov (2014), implying that, for these data, a valid rank order comparison cannot be made if the ML alignment method is used.

**Table 5:** (Non)invariant parameters for ML alignment optimization.

**Intercepts/Thresholds**	
Q12a	33 **(40) (56) (100) (124)** 152 191 203 **(208)** 246 **(250) (276) (376) (392) (410)** 428 440 484
	**(554) (578) (608)** 643 703 705 710 752 **(756)** 792 **(826) (840)**
Q12b	33 40 56 **(100)** 124 **(152) (191) (203)** 208 246 250 276 376 392 410 **(428) (440) (484)** 554
	578 **(608) (643) (703)** 705 **(710) (752)** 756 **(792) (826) (840)**
Q12c	33 **(40)** 56 **(100)** 124 **(152)** 191 **(203)** 208 **(246)** 250 276 376 **(392) (410) (428) (440)** 484
	**(554)** 578 **(608)** 643 703 705 **(710) (752) (756) (792) (826) (840)**
**Loadings**	
Q12a	33 40 56 100 124 152 191 203 208 246 250 276 376 392 410 428 440 484 554 578 608 643 703 705 710 752 756 792 826 840
Q12b	33 40 56 100 124 152 191 **(203)** 208 246 250 **(276)** 376 392 410 428 440 484 554 **(578)** 608 643 703 705 **(710)** 752 756 792 826 840
Q12c	33 40 **(56)** 100 124 152 191 203 208 246 250 276 376 392 **(410)** 428 440 484 554 578 608 643 703 705 710 752 **(756)** 792 826 840

*Noninvariant parameters are in bold and within parentheses*.

###  BAMI

For BAMI, only the results for the models that converged are presented here (models with a prior variance of 0.02, 0.01, 0.001, 0.0005, and 0.0001). These models also converged when the number of iterations was doubled, which was not the case for the models with other prior settings.

To select the model(s) with a good fit, one could use model fit indices, but just as with regular SEM there is not one single statistic that should be used, and only a combination of fit indices should be used to indicate model fit. Table 6 shows fit statistics for the models with prior variances 0.02, 0.01, 0.001, 0.0005, and 0.0001. Table 6 shows that only for models with a prior variance of 0.02 and 0.01 the PPP >0 (0.36 and 0.12, respectively) and the 95% CI contains 0. BRMSEA is 0.014 for the model with a prior variance of 0.02, and it is 0.049 for the model with prior variance of 0.01. For the other models, BRMSEA >0.1. For the model with prior variance 0.02, both BCFI and BTLI are 1.00, and for the model with prior variance 0.01 BCFI is 0.999 and BTLI is 0.994, indicating good model fit. Using PPP-value, the model with variance 0.02 comes closest to 0.5 with a PPP-value of 0.36. However, the PPP-value might be untrustworthy because of the large sample size of our study (24,583 respondents) (Asparouhov and Muthén, 2019). Hoijtink and van de Schoot (2018) stated that the PPP-value is not suitable for evaluating small priors. Concerning both CI and BRMSEA, only the models with prior variances of 0.02 and 0.01 indicate a good fit. When looking at BCFI and BTLI, however, the models with prior variances of 0.02, 0.01, 0.001, and 0.005 all indicate good fit, although fit statistics approach to their cutoff values as prior variances decline. When combining the above results with the DIC for the different models, the values for the model with prior variance 0.02 is the lowest (19,7428.86), indicating that this is the best fitting model based on *post-hoc* fit indices.

**Table 6:** Fit statistics of the BAMI models.

**Priorvariance**	**PPP**	**95% CI**	**BRMSEA**	**BCFI**	**BTLI**	**BIC**	**DIC**
0.02	0.363	[−52.706 to 79.836]	0.014	1.000	1.000	200288.68	197428.66
0.01	0.117	[−25.495 to 109.968]	0.049	0.999	0.994	200324.54	197514.18
0.001	0.000	[669.517 to 852.222]	0.117	0.976	0.968	201095.93	198186.01
0.0005	0.000	[1100.629 to 1286.848]	0.130	0.962	0.960	201546.82	198601.39
0.0001	0.000	[1873.721 to 2022.907]	0.148	0.938	0.949	202327.90	199334.78

*PPP, posterior predictive probability; CI, credibility intervals; BRMSEA, Bayesian root mean square error of approximation; BCFI, Bayesian comparative fit index; BTLI, Bayesian Tucker-Lewis index; BIC, Bayesian information criterion; DIC, deviance information criterion*.

**Figure 1:**
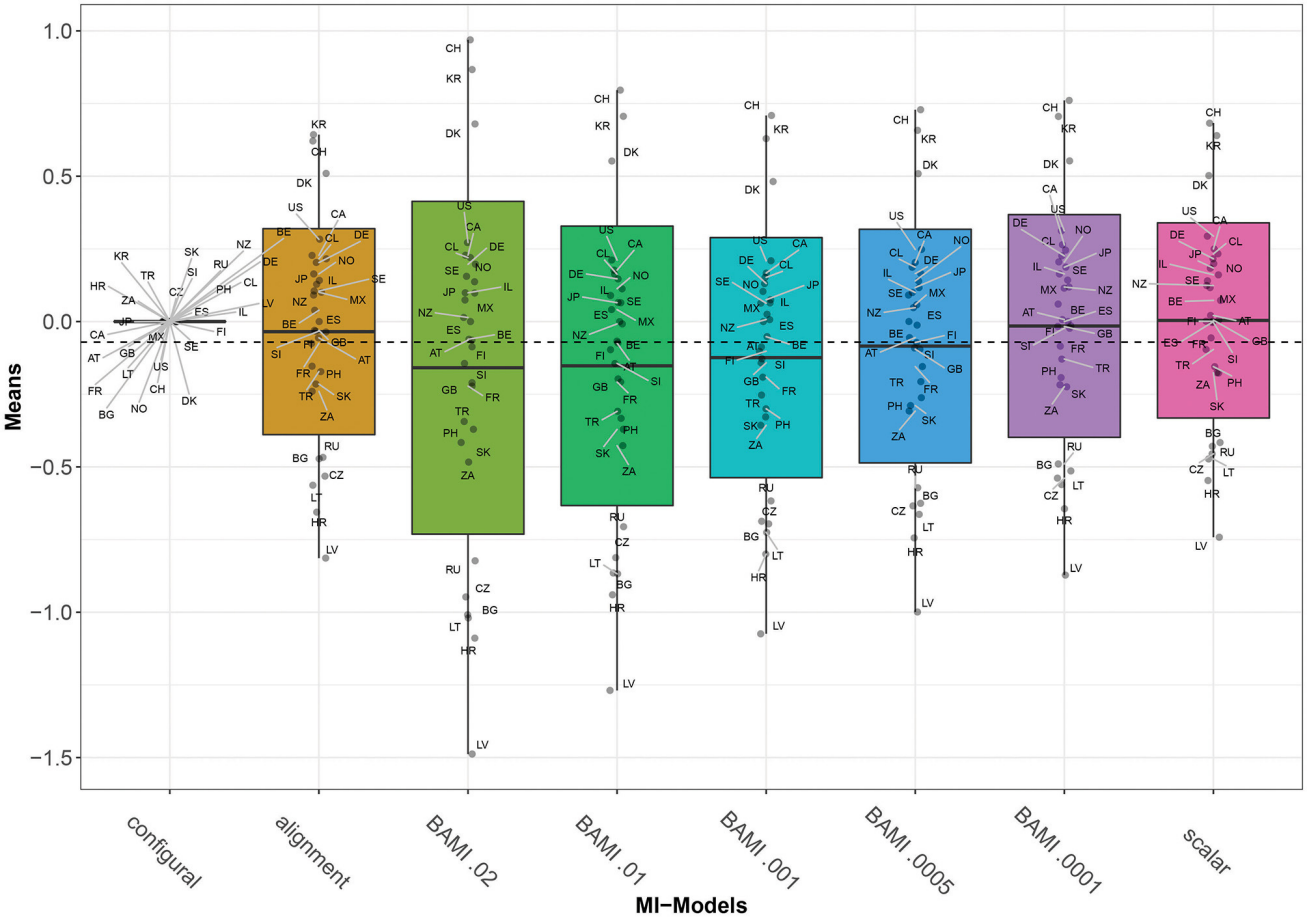
Means for configural invariance, scalar invariance, ML alignment, and BAMI models. AT, Austria; BE, Belgium; BG, Bulgaria; CA, Canada; CL, Chile; HR, Croatia; CZ, Czech Republic; DK, Denmark; FI, Finland; FR, France; DE, Germany; GB, Great Britain; IL, Israel; JP, Japan; LV, Latvia; LT, Lithuania; MX, Mexico; NZ, New Zealand; NO, Norway; PH, Philippines; RU, Russia; SK, Slovakia; SI, Slovenia; ZA, South Africa; KR, South Korea; ES, Spain; SE, Sweden; CH, Switzerland; TR, Turkey; US, United States. The x-axis shows the different models—configural, scalar, ML alignment, and BAMI—with their specific variances. The dashed black line shows the overall mean.

Figure 1 shows the means of the latent variable for the BAMI models with variances of 0.02, 0.01, 0.001, 0.0005, and 0.0001. From this figure it can be seen that with declining prior variance, the outcome of the model approaches that of the scalar model (on the right). This is to be expected, as the scalar model is a model of priors with a mean and variance of 0.

**Figure 2:**
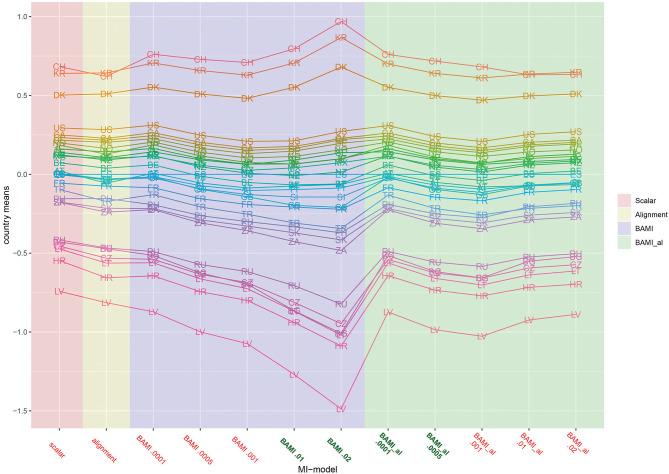
Means per country for scalar invariance, ML alignment, and BAMI models with and without alignment. AT, Austria; BE, Belgium; BG, Bulgaria; CA, Canada; CL, Chile; HR, Croatia; CZ, Czech Republic; DK, Denmark; FI, Finland; FR, France; DE, Germany; GB, Great Britain; IL, Israel; JP, Japan; LV, Latvia; LT, Lithuania; MX, Mexico; NZ, New Zealand; NO, Norway; PH, Philippines; RU, Russia; SK, Slovakia; SI, Slovenia; ZA, South Africa; KR, South Korea; ES, Spain; SE, Sweden; CH, Switzerland; TR, Turkey; US, United States. The x-axis shows the different models—scalar, ML alignment, and BAMI with and without alignment—with their specific variances. Models that appear to ba a good fit to the data are indicated in bold green, models with bad fit in red.

Figure 2 is a graph of the means per country per model (scalar invariance, ML alignment, and all BAMI models). For illustrative purposes, we present the results for BAMI both with and without alignment in one figure. Figure 1 shows that the overall mean differences between latent means of the different models are small but increase as the prior variance decreases: Δ_0.01−0.02_ is 0.007 and Δ_0.0005−0.0001_ is 0.069. For individual countries, this is not always the case: Figure 2 shows that, for the 15 lowest ranking countries this same pattern is visible, but for the top 15 countries the means increase with prior variance. However, as the countries rank lower, the differences between models increase. For the lowest-ranking country (Latvia) the difference between the model with prior variance 0.02 and that with prior variance 0.0001 is 0.616, while for the highest-ranking country (Switzerland) the difference is 0.208. For the three highest-ranking countries (Switzerland, South Korea and Denmark) the model with the highest prior variance (0.02) shows larger differences from the model with a prior variance of 0.01 (0.173, 0.161, and 0.128, respectively) than do other models with consecutively lower prior variances.

###  BAMI With Alignment

When the BAMI model with alignment is applied, first, the BAMI model is estimated. The outcome of the BAMI models is given in the description above (Table 6). From this BAMI model, a configural model is estimated, which is then aligned. This means that fit indices cannot be used to indicate model fit of the final model. Instead, just as with the ML alignment model, we use the percentage of non-invariant parameters to determine good model fit. Table 7 shows the number of non-invariant parameters per model.

**Table 7:** The number of non-invariant parameters for the BAMI models with alignment.

**Prior Variance**	**Number of non-invariant intercepts**				**Number of non-invariant loadings**				**Total number**	

	**Q12a**	**Q12B**	**Q12C**		**Q12a**	**Q12B**	**Q12C**		**Sum**	**%**
0.02	15	16	19		2	5	5		62	34.44
0.01	15	15	19		0	5	5		59	32.78
0.001	9	15	16		0	2	4		46	25.56
0.0005	4	11	12		0	1	2		30	16.67
0.0001	0	3	0		0	0	0		3	1.67

From this table, it can be seen that, as prior variances decrease, so does the number of non-invariant parameters. The three models with prior variances of 0.02, 0.01, and 0.001 all have a percentage of non-invariant parameters above 25% (although the model with prior variance 0.001 is only slightly above), making the results, and thus a group ranking from these models, unreliable. For the models with prior variances of 0.0005 and 0.0001 the percentages of non-invariant groups are 16.67 and 1.67, respectively, implying good model fit and a valid group ranking.

**Figure 3:**
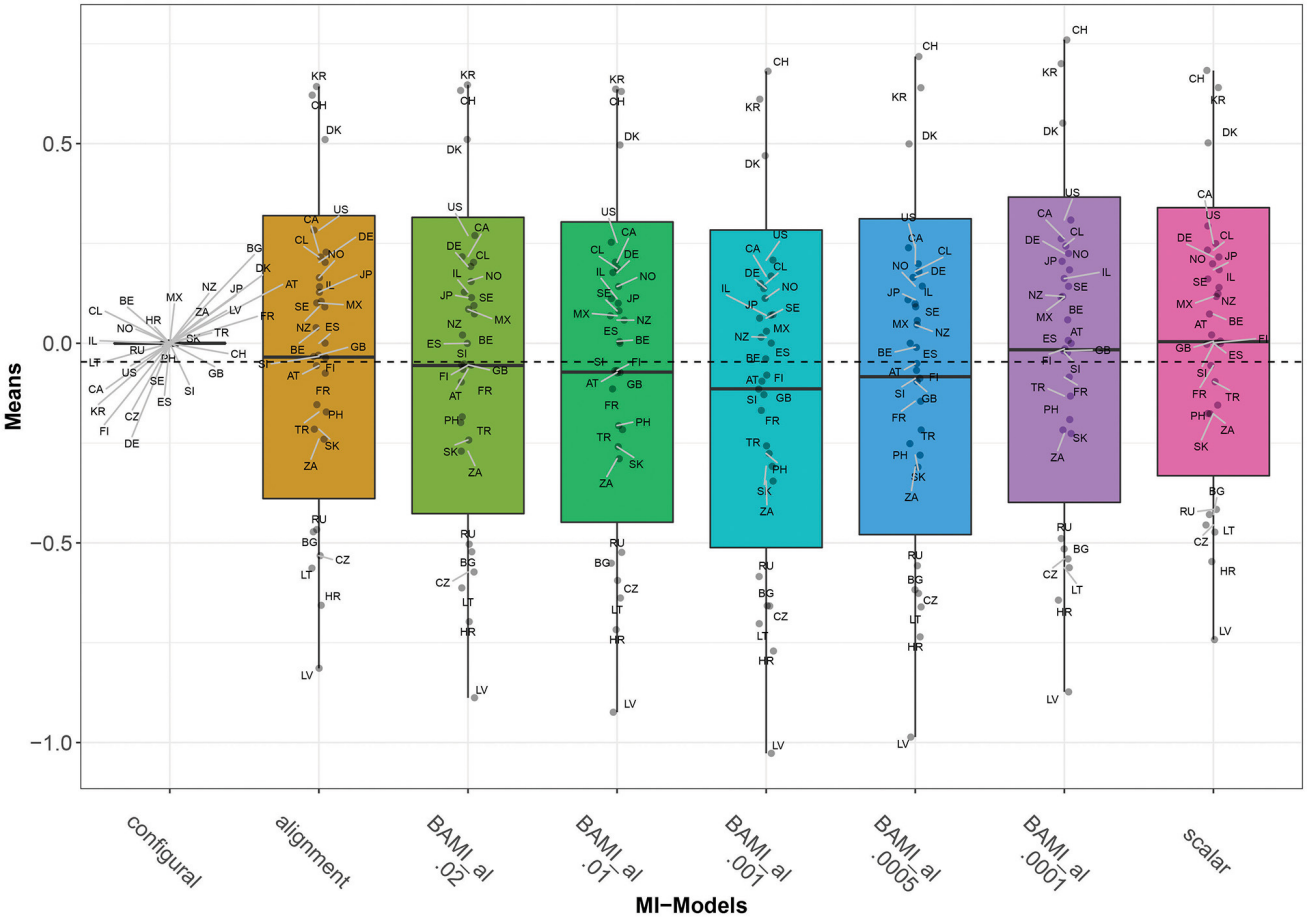
Means for configural invariance, scalar invariance, ML alignment, and BAMI models with alignment. AT, Austria; BE, Belgium; BG, Bulgaria; CA, Canada; CL, Chile; HR, Croatia; CZ, Czech Republic; DK, Denmark; FI, Finland; FR, France; DE, Germany; GB, Great Britain; IL, Israel; JP, Japan; LV, Latvia; LT, Lithuania; MX, Mexico; NZ, New Zealand; NO, Norway; PH, Philippines; RU, Russia; SK, Slovakia; SI, Slovenia; ZA, South Africa; KR, South Korea; ES, Spain; SE, Sweden; CH, Switzerland; TR, Turkey; US, United States. The x-axis shows the different models—configural, scalar, ML alignment, and BAMI with alignment—with their specific variances. The dashed black line shows the overall mean.

As with the ML alignment model, most non-invariant parameters are the intercept parameters. For the model with the lowest number of non-invariant parameters (prior variance 0.0001), these parameters belong to the intercepts of question 12b (are you willing to pay higher taxes to save the environment), for the countries Lithuania, South Africa, and Turkey. When taking into account only the models with a percentage of non-invariant parameters below 25%, there are 12 countries for which all parameters are invariant for both models: Spain, Austria, Canada, Denmark, Finland, Germany, Israel, Mexico, Russia, Slovakia, Slovenia, and Sweden. Figures 2, 3 show that, as the priors decrease, so do the mean differences of the model outcomes (both the overall means and the means per country).

Figure 3 shows that for the first three models with decreasing prior variance, the overall means also decrease. However, as prior variances decrease further (0.0005 and 0.0001), they rise slowly toward the means of the scalar model. The differences between means of models with consecutive priors are less clear than for the BAMI models (Figure 1). Now, Δ_0.01−0.02_ 0.016, Δ_0.001−0.01_ 0.042, Δ_0.0005−0.0001_ 0.0675, and Δ_0.001−0.0005_ 0.031. Although this pattern is visible in the means per model (Figure 3), it is less distinctive when looking at the means of individual countries (Figure 2). In that case, this pattern is most pronounced for Latvia and, to a lesser extent, Bulgaria, Lithuania, Hungary, Russia, South Africa, Slovakia, Turkey, the Philippines, Denmark, Croatia, and Switzerland. Figures 2, 3 show that, as prior variances decrease, so do the mean differences of the model outcomes (both the overall means and the means per country). Again, Latvia is the country with the most pronounced differences when comparing different priors.

###  Ranking

**Figure 4:**
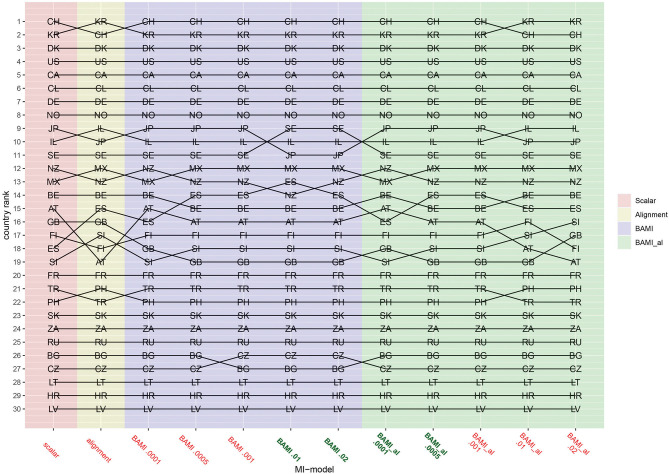
Rankings per model. AT, Austria; BE, Belgium; BG, Bulgaria; CA, Canada; CL, Chile; HR, Croatia; CZ, Czech Republic; DK, Denmark; FI, Finland; FR, France; DE, Germany; GB, Great Britain; IL, Israel; JP, Japan; LV, Latvia; LT, Lithuania; MX, Mexico; NZ, New Zealand; NO, Norway; PH, Philippines; RU, Russia; SK, Slovakia; SI, Slovenia; ZA, South Africa; KR, South Korea; ES, Spain; SE, Sweden; CH, Switzerland; TR, Turkey; US, United States. The x-axis shows the different models—scalar, ML alignment, and BAMI with and without alignment—with their specific variances. Models that appear to ba a good fit to the data are indicated in bold green, models with bad fit in red.

Figures 1–3 show that the latent means vary depending on the choice of prior variance. Models with smaller prior variances seem to have outcomes that approach the outcome of the scalar model. However, there is some variation at the country level. From Figure 2, we observe that there appear to be four different groups of countries with similar means: Switzerland, South Korea and Denmark at the top, then a large group with the United States, Canada, Chile, Germany, Norway, Japan, Israel, Sweden, New Zealand, Mexico, Austria, Great Britain, Finland, Spain, Slovenia, France, Turkey, Philippines, Slovakia, and South Africa. The third group comprises Russia, Czech Republic, Bulgaria, Lithuania, and Croatia, while the bottom group consists of only one country: Latvia. In particular for the second group, means are very close together, and it can be difficult to distinguish individual country means. Figure 4 shows the ranking of the 30 countries for the analyzed models that converged. This figure shows that for nearly all the models, ranking changes somewhat when a different prior variance is used. Upon closer inspection, 13 of the 30 countries occupy the same place in the ranking for all the models, and most changes appear to be in the middle of the ranking. When combined with Figure 2, it becomes clear that country mean differences are small, especially for the BAMI model with alignment. For the BAMI models, country means differ slightly more, especially at the top and the bottom of the ranking. Mean differences for Latvia decrease with decreasing prior variance, and these differences are larger than the overall mean difference per model.

Comparing the individual country means of the BAMI and BAMI with alignment model shows that for all countries the differences between the models decrease with prior variance: differences between models are lowest when models with the lowest prior variances are compared. For the models with a prior variance of 0.0001 the country rankings are the same.

Focusing on only the models that appear to have a good fit to the data, according to their fit statistics, only the BAMI models with a prior variance of 0.02 and 0.01 and BAMI models with alignment with a prior variance of 0.0005 and 0.0001 are of importance. When comparing the rankings of these models, the ranking for the BAMI models is almost identical: only Spain and New Zealand switch places when changing models. The ranking of the BAMI models with alignment shows more variation: 23 countries rank the same for both models, while Mexico, New Zealand, Belgium, Austria, Great Britain, and Slovenia all shift one place up or down and Spain moves two places in the ranking.

These figures show that, regardless of prior variance or even model fit, people in Switzerland and South Korea are most motivated to sacrifice for the environment, while people in Bulgaria and Latvia are less motivated to sacrifice for the environment.

#### Decision Tree

**Figure 5:**
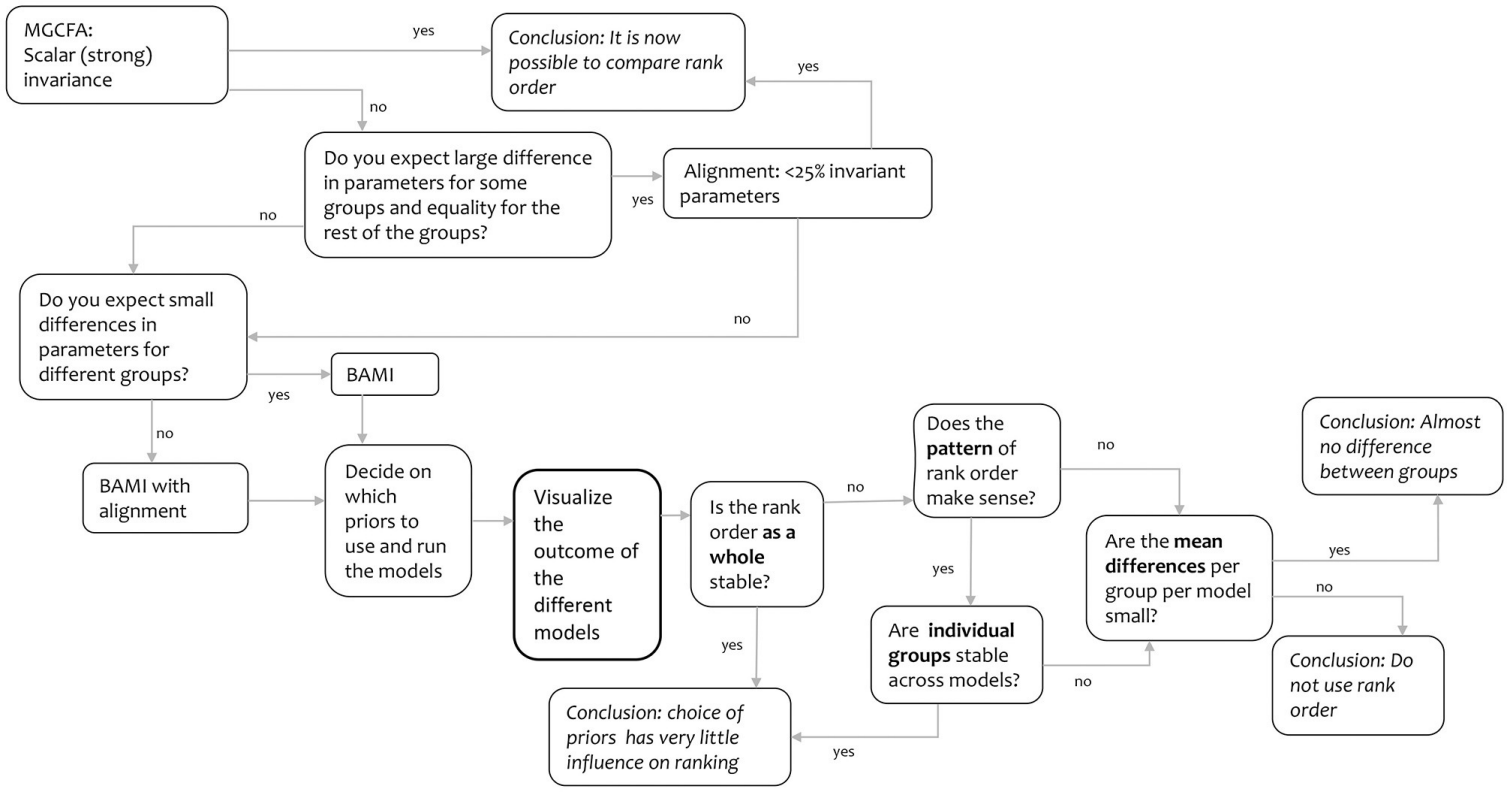
Decision tree.

As it can be difficult to draw conclusions from the means and rankings as shown in Figures 1–4, we devised a decision tree (Figure 5). This tree provides some insight into the decisions that we had to make regarding group means, group rankings and the influence of priors. Based on this decision tree, other readers might come to different conclusions. The tree comprises the entire process needed to evaluate the information contained in Figures 1–4, starting with the MGCFA test for scalar invariance:

1. We started with an ML MGCFA test for scalar invariance. To test for scalar invariance it is necessary that configural and metric invariance are met.
  a. Yes: It is now possible to compare ranks.
  b. No: Try another method to make means comparison valid. Go to step 2.
    We did not find scalar invariance, so we followed the “no” arrow to step 2.

2. Do you expect a large difference in parameters for some groups and equality for the rest of the groups^5^?
  a. Yes: Equality for almost all groups. Go to step 3.
  b. No: Only small differences or small and large differences. Go to step 4.
    We assumed there would be some differences in the parameters, although we did not know how large these differences would be or how many groups would differ from each other, so we first followed the “yes” arrow to step 3 and tested for ML alignment.

3. Does the alignment optimization yield < 25% non-invariant parameters?
  a. Yes: The rank order can be trusted
  b. No: Try another method to make means comparison valid. Go top step 4.
    This yielded >25% non-invariant parameters, so we followed the “no” arrow to step 4.

4. Do you expect only small differences in parameters for different groups^6^?
  a. Yes: Only small differences. Use BAMI, Go to step 4A.
  b. No: Both small and large differences. Use BAMI in combination with alignment optimization. Go to step 4B.
    Now, we had to decide whether we expected small or large differences in parameters for different groups. Since we did not know how large the differences per group were, we used both, as they lead to step 5 in this decision tree; which option we chose would not make a difference.

5. Then, we needed to decide which priors to use and run the different models. We based our priors on previous literature on the use of BAMI (both simulations and empirical examples). We then moved to step 6.
6. We visualized the outcomes of the different models [scalar, ML alignment, and BAMI models (with and without alignment) that converged] in Figures 1–4 (means and group rankings). The code to create these Figures can be found in Appendix C on Arts et al. (2021). We moved to step 7.
7. Is the rank order **as a whole** stable? (Figure 4)
  a. Yes: No or only minor changes in rank. Then the rank order is not at all or only slightly influenced by the choice of priors.
  b. No: Many changes across groups and models. Go to step 8.
    Since there were numerous changes in the rank order, we did not consider the rank order stable and we followed the “no” arrow to step 8.

8. Does the pattern of the rank order across different models make sense?
  a. Yes: Many changes, but all changes in the same section of the ranking (e.g., top) or the same groups change rank. Go to step 9.
  b. No: The pattern seems erratic. Go to step 10.
    From Figure 5 we concluded that the upper and the lower parts of different rankings hardly change, and most rank changes take place in the middle part of the ranking. We considered this a logical pattern and followed the “yes” arrow.

9. Are individual groups stable across models?
  a. Yes: Individual groups never move more than one place up or down in the ranking across different models. Then the rank order is not or only slightly influenced by the choice of priors
  b. No: Individual groups continue moving up or down the ranking across different models.
    Changes in rank nearly always applied to the same countries, making the pattern rather stable. However, as some countries moved up or down two or three positions in the ranking across models, we found that stability of the groups could not be guaranteed. We followed the “no” arrow.

10. Are the mean differences per group per model small?
  a. Yes. There is almost no difference between groups, and the influence of the priors is small.
  b. No: Do not use rank order.
    Figure 3 shows that the differences per group are quite small, especially in the middle part of the ranking where most changes in rank take place. We therefore conclude that there is almost no difference between groups.

## Conclusion and Discussion

The latent variable “willingness to sacrifice for the environment” (WTS) is an important aspect of environmental concern. It can provide insights into the intentional behavior regarding environmental concern, which, in turn, provides more insight into the willingness of the respondents to take action to protect the environment. Given that country rankings of latent means of WTS are frequently used in comparative studies, it is important to assess whether substantive findings are indeed trustworthy or are methodological artifacts due to lack of metric or scalar invariance. The latent variable WTS was, in combination with the latent variable environmental attitude (EA), previously tested for MI by Mayerl and Best (2019). Using MGCFA, they did not find scalar invariance, questioning comparisons of the latent means across countries. However, recent discussions in MI point out that the approach of ML MGCFA may be too strict, and approaches such as alignment or BAMI, or a combination of both, may be a viable solution when exact scalar invariance tests fail (e.g., van de Schoot et al., 2013; Asparouhov and Muthén, 2014). In this article, we examined WTS in 30 different countries, using the 2010 ISSP data. We did not establish scalar invariance when using MGCFA, which is in line with the findings of Mayerl and Best (2019). In addition to MGCFA, we also assessed MI using ML alignment, BAMI and BAMI with alignment method.

Based on our results, we can determine which countries consistently rank high on the latent variable WTS (Switzerland and South Korea) and which countries consistently rank low (Latvia). However, we cannot say that, e.g., respondents in Sweden are more or less willing to sacrifice for the environment than respondents in Mexico. Thus, a more general conclusion about these country rankings can be drawn (high, low), but when exact ranking (e.g., fourth or fifth), or even exact means, are important, these country rankings should not be used. In conclusion, only with BAMI plus alignment optimization we were able to obtain stable results. From these, we can conclude that people in Switzerland and South Korea are most motivated to sacrifice for the environment, while people in Latvia are less motivated to sacrifice for the environment.

Regarding the use of different prior variances when using the BAMI method, models with a prior variance of 0.02 and 0.01 showed good model fit for most fit statistics (PPP, 95% CI, BRMSEA, BCFI, BTLI). For the model with variances of 0.001 and 0.0005 BCFI and BTLI were within limits. When taking into account that PPP might incorrectly identify model fit for models with large sample sizes (van de Schoot et al., 2012; Mulder, 2014), the results of BAMI models with a variance of 0.001 and 0.0005 might still fit the data. BIC and DIC are lowest for the BAMI model with a prior variance of 0.02, but the use of DIC for models with small prior variances has been disputed by Hoijtink and van de Schoot (2018). For the BAMI models with alignment the models with the smallest prior variances (0.0005 and 0.0001) give trustworthy results with a percentage of non-invariant parameters of 16.67 and 1.67%, respectively. This indicates that the BAMI models with a prior variance of 0.02 and 0.01 are a good fit to the data, while for the BAMI with alignment models, the models with a prior variance of 0.0001 and 0.0005 give trustworthy results.

Concerning comparing means of the BAMI models with different prior variances, both with and without alignment, the means are very similar for the models with prior variances of 0.0001 (difference of the overall mean per model is 0.001). These country rankings are, with the exception of Great Britain and Spain (rank 16 and 18, respectively) also the same for the scalar model. However, the scalar model and the BAMI model with a prior variance of 0.0001 cannot be assumed to be a good fit to the data (see Tables 4, 6), while the BAMI model with alignment with the same prior can. When comparing two models that indicate reliable outcomes—the BAMI with prior variance of 0.02 and the BAMI with alignment model with a prior variance of 0.0001—differences per country are much larger (ranging from 0.616 to 0.029, with the exception of 0 for Spain), thus indicating that prior variances can have a large influence on model outcomes, and that the model results that appear to be reliable, can be very close or even equal to the results of a model that should be rejected. This also shows the difficulty of comparing BAMI models to BAMI models with alignment: because the ground on which models should be rejected are very different, it is difficult to say which model should be preferred, if any.

It is our opinion that visualizing the results facilitates determining of the effect of different prior variances. A visual presentation of the results could be a valuable addition to the presentation of results in elaborated tables that can be challenging to interpret, especially when many groups are compared. The visualization approach that we used in this article is, however, not the only possibility to visualize (MI) results. Depending on e.g., research question, group size, and personal preferences, the researcher can choose other ways to visualize the results. For example, van de Schoot et al. (2015) chose to display the effect of different prior variances on the differences between groups in several line charts, while Pokropek et al. (2020) decided to use color-coded tables to identify the most suitable fit statistic to identify the optimal prior, Zercher et al. (2015) used scatter plots to represent latent means per country, and van de Vijver et al. (2019) used a 3-dimensional plot showing the Euclidean distances between different groups. However, most researchers still use (mainly) tables to present their results (e.g., Chiorri et al., 2014; De Bondt and Van Petegem, 2015; Gucciardi et al., 2016; Seddig and Leitgöb, 2018; Solstad et al., 2020; Vilar, 2020). We propose that a visual presentation of the results can improve the comprehension of test results, and can serve as a useful addition to previous presentations of results. This can be particularly useful when a researcher is faced with contradictory priors: a visualization of model outcomes with these different priors immediately shows the effect that the priors might or might not have. In particular researchers who are less familiar with the subject of Bayesian modeling might benefit from such a visual presentation.

###  Limitations

This study has some limitations, that need to be mentioned. First, the latent variable WTS is linked to intentional behavior, but intentional behavior alone cannot explain environmental concern. This one-scale, three-item model is an oversimplification of real-world data: multiple latent variables are required to provide insight into environmental concern. If MI holds for WTS in combination with other latent variables (e.g., EA) a country ranking would be more meaningful when determining nationwide environmental concern. Also, WTS is mainly financially driven (two out of three question refer to paying to protect the environment: see Table 1). This would mean that respondents who cannot or do not want to contribute financially but are willing to contribute in some other way affect this latent variable differently than those who are willing to sacrifice financially. Second, in the analysis of the different methods, most settings were Mplus default settings. Using different settings might lead to different outcomes: e.g., a different simplicity function when using alignment could affect model outcome when the alignment optimization is used. It is also possible to choose Bayes as an estimator instead of ML. In that case, analysis starts with the same M_0_ model as for ML alignment, and then loadings and intercepts are estimated using noninformative priors using Equation (3) (Asparouhov and Muthén, 2014). For other Mplus settings see Muthén and Muthén (2019). However, testing the many different settings in Mplus is beyond the scope of this article. Third, we used a decision tree (Figure 5) to interpret the results based on a visual inspection of country means and rankings. In this decision tree, several choices have to be made based on the ranking order and pattern (Figure 4). This pattern might not be interpreted by everyone in the same way: at Step 7 (is the rank order as a whole stable), Step 8 (does the pattern of the rank order make sense), and Step 9 (are individual groups stable across models) the reader has to decide whether a pattern is stable, the rank order makes sense, and individual groups are stable across models. It is also up to the reader to decide if the differences between group means are small or large (step 10). So, depending on the reader, conclusions might be different. However, we argue that using a decision tree always involves arbitrary decisions, like non-testable identification constraints, see for a discussion Little et al. (2006). We also believe that, in this case, the benefits of a decision tree (transparency to the workflow) outweigh the disadvantages.

###  Future Research

In this article we show that, for WTS, MI is present, making a ranking of countries possible. We also show that country means are not independent of specified priors and that, although the differences are small, an exact country ranking cannot be assumed. A combination of multiple latent variables (EA, knowledge of environmental concern, risk perception) might provide more insight into environmental concern. This would complicate model specification and analysis somewhat, since it would, e.g., make the use of priors on cross-loadings an important part of the model (Xiao et al., 2019; Liang et al., 2020). Another potential promising area of future study is the method of Robitzsch (2020), which improves the alignment optimization. A comparison with BAMI has not been made, yet. Future research may provide more insight into WTS and the topic of environmental concern. Looking at Figure 2, we see that four groups of countries have very similar means. It would be interesting to further investigate why this division into four groups appears. Is this also the case when other latent variables are investigated (separately or combined with WTS)? Is it purely data driven or are there underlying reasons that can explain these four groups (psychological, sociological, political, economic, etc.)? A multilevel model that includes such factors, might shed more light on why these four groups exist. The prior variances that we used in this article were based on previous literature on prior selection (both simulation and empirical studies). Another approach to selecting priors could be to consult literature on environmental change to determine the factors that drive environmental concern. Priors could then be based on e.g., socioeconomic status of a country, geography (and thus the environmental threat a particular country faces) or political system and stability. Multilevel tests aiming to unravel these factors have been conducted by e.g., Marquart-Pyatt (2012a); Fairbrother (2013); Pampel (2014), and Pisano and Lubell (2017), but, to our knowledge, such factors have not yet been included in a Bayesian model. However, the use of such priors would emphasize the strength of Bayesian modeling.

## Data Availability Statement

Publicly available datasets were analyzed in this study. This data can be found at: doi: 10.4232/1.13271. All appendices, the scripts to reproduce our results, the final output files and additional material can be found on website of the Open Science Framework (OSF): https://osf.io/mvyws/ (Arts et al., 2021).

## Author Contributions

IA, RvdS, and KM designed the study and wrote the article. IA conducted the analyses. QF provided the necessary code for visualization. All authors contributed to the article and approved the submitted version.

## Conflict of Interest

The authors declare that the research was conducted in the absence of any commercial or financial relationships that could be construed as a potential conflict of interest.

## Footnotes

^1^Residual invariance means that the sum of specific variance (variance of the item that is not shared with the factor and error variance) are also equal across groups (Putnick and Bornstein, 2016). Since this is not a requirement for comparing means across groups, we do not report it in this article.

^2^In previous research, the term Approximate MI (van de Schoot et al., 2013) has sometimes been used as a collective term for any method that can be used when the criteria for the exact scalar model are not fulfilled (Russell et al., 2016; Flake and McCoach, 2018), and sometimes to mention a specific method (e.g., Byrne and van de Vijver, 2017; Amérigo et al., 2020). To prevent any further confusion, we propose to use the term Bayesian Approximate Measurement Invariance (BAMI) when using a Bayesian model with strong informative priors on differences between factor loadings and/or intercepts, thus excluding non-Bayesian (ML or empirical Bayes) type of methods like random item effects (Fox and Verhagen, 2018).

^3^Technically speaking, this is not entirely correct: for identification of the model, Mplus by default fixes the loading/intercept of the first item of every group to 1. For more details about parameterization of CFA models we refer the interested reader to Little et al. (2006).

^4^Hoijtink and van de Schoot (2018) demonstrated that, with increasing sample sizes, the PPP-value does not decrease, but increases. Therefore, the prior-posterior predictive *p*-value (PPPP-value) was proposed by Hoijtink and van de Schoot (2018), and a generalized version was implemented in Mplus by Asparouhov and Muthén (2017). Whereas, the original PPP-value is a test of model fit which tests the fit of the model to the data and is based on comparing the model with the unrestricted covariance model, the PPPP-value is a test for the approximate parameters in the model. The PPPP-value is not a test for model fit and should not be interpreted as evidence that the model fits the data. The proper interpretation of the PPPP-value is given by Asparouhov and Muthén (2017): “If the test does not reject, the minor parameters (represented by *θ*_1_) can be assumed to come from N(0, v) distribution, with v being a small variance. More broadly speaking, if the PPPP does not reject, that means that there is no evidence in the data for the minor parameters in model M(*θ*_1_, *θ*_2_) to be outside the N(0, v) distribution” (p. 10). Here, *θ*_2_ represents the large parameters of model M. Unfortunately, the PPPP-value is not yet available for BAMI in Mplus.

^5^When in doubt whether large difference in parameters for some groups are to be expected, it is advisable to consult a substantive expert of your field. The decision whether large parameters can be expected should be based on previous research and/or expertise.

^6^Similarly, when in doubt whether small differences for many groups are to be expected, it is advisable to consult a substantive expert of your field. The decision whether small parameter differences can be expected should be based on previous research and/or expertise.
